# Molecular Cytogenetic and Y Copy Number Analysis of a Reciprocal ECAY-ECA13 Translocation in a Stallion with Complete Meiotic Arrest

**DOI:** 10.3390/genes12121892

**Published:** 2021-11-26

**Authors:** Caitlin Castaneda, Agustin J. Ruiz, Ahmed Tibary, Terje Raudsepp

**Affiliations:** 1 Department of Veterinary Integrative Biosciences, Texas A&M University, College Station, TX 77843, USA; ccastaneda@cvm.tamu.edu; 2 Department of Veterinary Clinical Sciences, Washington State University, Pullman, WA 99164, USA; agustin.ruiz@wsu.edu (A.J.R.); tibary@wsu.edu (A.T.)

**Keywords:** horse, FISH, meiotic executioner genes, MCSI, azoospermia, Y-autosome translocation, Friesian breed

## Abstract

**Simple Summary:**

We present a detailed molecular cytogenetic analysis of a translocation between horse (ECA) chromosomes Y and 13 in a Friesian stallion with complete meiotic arrest and azoospermia. We use two-color fluorescence in situ hybridization with select ECAY and ECA13 markers and show the location of translocation breakpoints in ECAY and in ECA13. The translocation generates two abnormal chromosomes: one comprised of the short arm of ECA13 and the proximal gene-poor 2/3 of ECAY and another, the long arm of ECA13 and the distal 1/3 of ECAY containing most of the single copy and ancestral genes. A copy number (CN) analysis of select ECAY multicopy genes shows that the Friesian stallion has significantly reduced CNs of *TSPY*, *ETSTY1,* and *ETSTY5*, suggesting some genetic loss due to the translocation. We discuss likely meiotic behavior of abnormal chromosomes and theorize about the possible effect of the aberration on Y regulation and the progression of meiosis. The study adds a unique case to equine clinical cytogenetics and contributes to understanding the role of the Y chromosome in male meiosis.

**Abstract:**

We present a detailed molecular cytogenetic analysis of a reciprocal translocation between horse (ECA) chromosomes Y and 13 in a Friesian stallion with complete meiotic arrest and azoospermia. We use dual-color fluorescence in situ hybridization with select ECAY and ECA13 markers and show that the translocation breakpoint in ECAY is in the multicopy region and in ECA13, at the centromere. One resulting derivative chromosome, Y;13p, comprises of ECAY heterochromatin (*ETSTY7* array), a small single copy and partial Y multicopy region, and ECA13p. Another derivative chromosome 13q;Y comprises of ECA13q and most of the single copy ECAY, the pseudoautosomal region and a small part of the Y multicopy region. A copy number (CN) analysis of select ECAY multicopy genes shows that the Friesian stallion has significantly (*p* < 0.05) reduced CNs of *TSPY, ETSTY1,* and *ETSTY5*, suggesting that the translocation may not be completely balanced, and genetic material is lost. We discuss likely meiotic behavior of abnormal chromosomes and theorize about the possible effect of the aberration on Y regulation and the progression of meiosis. The study adds a unique case to equine clinical cytogenetics and contributes to understanding the role of the Y chromosome in male meiosis.

## 1. Introduction

Translocations between the Y chromosome and an autosome are rare in humans and animals. In the general human population, based on a study of 11,148 newborn infants, the incidence of Y-autosome translocations is approximately 1/2000 [1]. In domestic animals, five cases have been reported in pigs [2,3,4,5], two cases in cattle [6,7], and one case in horses [8].

The phenotypes associated with Y-autosome translocations are heterogeneous, vary within and between species and depend on several factors. Interspecific variation is largely due to the known species’ differences in the genetic content and organization of the Y chromosome [9,10]. It also matters which region of the Y chromosome is involved, whether the associated autosome is an acrocentric or a non-acrocentric, and whether the translocation is reciprocal or non-reciprocal. For example, in humans, non-reciprocal translocations of the distal heterochromatic portion of Yq to an acrocentric autosome does not affect the phenotype or fertility and has been transmitted as chromosomal variants both by men and women [11,12]. In contrast, 80% of men with balanced reciprocal Y-autosome translocations involving euchromatic portions of the Y have non-obstructive oligozoospermia or azoospermia [11,13,14]. The few Y-autosome translocations described in pigs have all been reciprocal, involving both acrocentric and non-acrocentric autosomes, and all resulted in azoospermia [2,3,4,5]. In cattle, on the other hand, where all autosomes are acrocentric [15], one case of a reciprocal Y-autosome translocation had azoospermia [7], and the other showed normal libido and sex development [6].

The genetic content of the Y chromosome and the type of the autosome involved in Y-autosome translocations directly affect the meiotic behavior of the aberrant chromosome and the functional status of both the Y-linked and autosomal genes [3,5,13]. In normal meiosis prophase I, autosomal homologs synapse, recombine and remain transcriptionally active. The sex chromosomes, the Y and the X, on the other hand, are not homologs and synapse only across the small pseudoautosomal region [16]. Due to this, for the normal progression of male meiosis, the sex chromosomes must undergo transcriptional silencing, known as meiotic sex chromosome inactivation (MSCI) [17]. Meiotic studies in human [13] and pig [3,5] show that Y-autosome translocations can negatively affect autosomal synapsis and MSCI, resulting in transcriptional silencing of autosomal genes and upregulation of Y-linked genes, leading to meiotic arrest and azoospermia. These studies clearly show that for better understanding of the genetic consequences of Y-autosome translocations in different species, conventional cytogenetic findings should be combined with molecular cytogenetic and functional analyses and interpreted in the context of Y chromosome organization of the species in question.

We recently presented a detailed clinical characterization of the first case of Y-autosome translocation in horses, a Friesian stallion with complete azoospermia and a reciprocal translocation rcp (Y;13) [8]. Cytogenetic characterization of this case was limited to G- and C-banding and fluorescence in situ hybridization (FISH) to confirm that the translocation was reciprocal. The aim of the present study is to conduct a detailed molecular cytogenetic analysis of the rearrangement to determine translocation breakpoints and evaluate the genetic content of aberrant chromosomes. The possible effect of the aberration on Y chromosome regulation and meiotic progression is discussed.

## 2. Material and Methods

### 2.1. Animal and Samples

Sodium heparin- and EDTA-stabilized blood samples were obtained from the previously described azoospermic Friesian stallion (case ID H787) with a cytogenetically confirmed Y;13 reciprocal translocation [8].

### 2.2. Chromosome Preparations for Molecular Cytogenetic Analysis

Chromosome preparations were obtained from short-term blood lymphocyte cultures following standard procedures described elsewhere [18]. Briefly, 1 mL of sodium heparin-stabilized peripheral blood was mixed with 9 mL culture medium containing RPMI-1640 with Glutamax (Gibco), 30% fetal bovine serum (R&D Systems Inc., Minneapolis, MN, USA), 1× antibiotic-antimycotic (Invitrogen, Waltham, MA, USA), and 1.4 µg/mL pokeweed mitogen (Sigma Aldrich, St. Louis, MO, USA). The cultures were grown for 72 h, harvested with demecolcine solution (final conc. 0.1 µg/mL; Sigma Aldrich), treated with optimal hypotonic solution (Rainbow Scientific, Windsor, CT, USA), and fixed in 3:1 methanol:acetic acid. Chromosome preparations were performed on clean wet slides, air dried and stored at −20 °C until needed.

### 2.3. Selection of Probes for Fluorescence In Situ Hybridization (FISH)

CHORI 241 (CH241) Bacterial Artificial Chromosome (BAC) library (https://bacpacresources.org/, accessed on 18 September 2021) clones spanning horse (*Equus caballus*, ECA) chromosome 13 (ECA13) were identified from the CH241 genomic clone track of the horse reference genome EquCab3 in NCBI Genome (https://www.ncbi.nlm.nih.gov/genome/, accessed on 18 September 2021) or from the integrated physical map of the horse genome [19]. Information for horse Y chromosome BAC clones was retrieved from the BAC tiling path of the ECAY sequence map [9] and BACs corresponding to the pseudoautosomal region (PAR) from the horse PAR BAC tiling path [20]. The summary of information about the BAC clones used for FISH in this study is presented in Table 1. In addition to BACs, we used a biotin-labeled microdissected ECA13-specific painting probe [21].

### 2.4. Genomic and BAC DNA Isolation

Genomic DNA (gDNA) was isolated from EDTA-stabilized peripheral blood using the Gentra Puregene Blood Kit (Qiagen, Hilden, Germany) following the manufacturer’s protocol. The DNA was checked for quality and quantity with the Nanodrop 2000 spectrophotometer (Thermo Scientific, Waltham, MA, USA). ECA13, ECAY, and PAR BAC clones were picked from the CHORI 241 BAC library (https://bacpacresources.org/, accessed on 18 September 2021). The BACs were grown overnight in 100 mL 2YT (Life Technologies, Carlsbad, CA, USA) supplemented with 30 mg/mL chloramphenicol (Sigma Aldrich) and BAC DNA was isolated using the Plasmid Midiprep kit (Qiagen).

### 2.5. Fluorescence In Situ Hybridization (FISH)

Two-color FISH was performed according to our standard protocol [18] using differently labeled combinations of two or three probes. The probes were labeled by nick-translation either with biotin or digoxigenin using the BIO- or DIG-Nick Translation Mix (Roche, Basel, Switzerland), respectively. Biotin-labeled probes were detected with avidin-Alexa Fluor 488 (Invitrogen) and dig-labeled probes with anti-digoxigenin-Rhodamine (Roche). The results were analyzed with a motorized fluorescence microscope Axio Imager M2p (Zeiss, Jena, Germany), equipped with the Isis v5.2 (MetaSystems GmbH, Altlußheim, Germany) software package for FISH analysis. A minimum of 20 metaphases were captured and analyzed for each experiment.

### 2.6. Digital Droplet PCR Analysis (ddPCR)

Digital droplet PCR assays were designed for eight multicopy genes in the male-specific region of horse Y chromosome (MSY) and an autosomal control gene *MYOZ1* (Table 2). Primers were designed with the Primer3 software [22] using reference sequences for horse MSY [9] and EquCab3 [23] so that the size of PCR products was in the range of 75–200 bp. Fluorescently labeled (FAM for MSY genes, VIC for autosomal *MYOZ1*) hydrolysis probes (TaqMan) were designed with the PrimerQuest™ tool (Integrated DNA Technologies (Clareville, IA, USA). The template gDNA was cleaved with EcoRI (Invitrogen) or NspI (New England Biolabs, Ipswich, MA, USA) restriction enzymes into <5 kb fragments to fit into individual droplets. The restriction enzyme chosen for the experiment was dependent on the MSY gene sequence. The ddPCR reactions were carried out on C1000Touch (Bio-Rad, Hercules, CA, USA) platform in 25 µL volume containing (final concentration) 1× ddPCR Supermix for Probes no-UTP, 10 µM forward and reverse primers for an MSY gene and the control gene, 250 nM TaqMan probe for an MSY gene and the control gene, one of the two restriction enzymes (diluted 1:1 in water), and 1–10 ng of undigested gDNA as a template. Droplets were generated using the QX200™ (Bio-Rad) automated droplet generator and the manufacturer’s protocol. The cycling parameters were conducted using the recommended protocol for performing genomic enzymatic digestion during the PCR experiment. The PCR plate was transferred to the QX200™ (Bio-Rad) droplet reader and the data were analyzed using the associated Quantasoft software v 1.7.4. The results are presented as number of copies per µL of the final 1× ddPCR reaction. The results were compared with previously available MSY copy number data for 16 normal control male horses [24].

A statistical analysis of gDNA copy number variations between the Friesian stallion (case ID: H787) and the control cohort (*n* = 16) was conducted using JMP v15 (JMP^®^, Version 15. SAS Institute Inc., Cary, NC, USA). A one-way Anova was used to generate F-statistic *p*-values.

## 3. Results

### 3.1. Molecular Cytogenetic Analysis of ECAY and ECA13 Reciprocal Translocation

**Figure 1:**
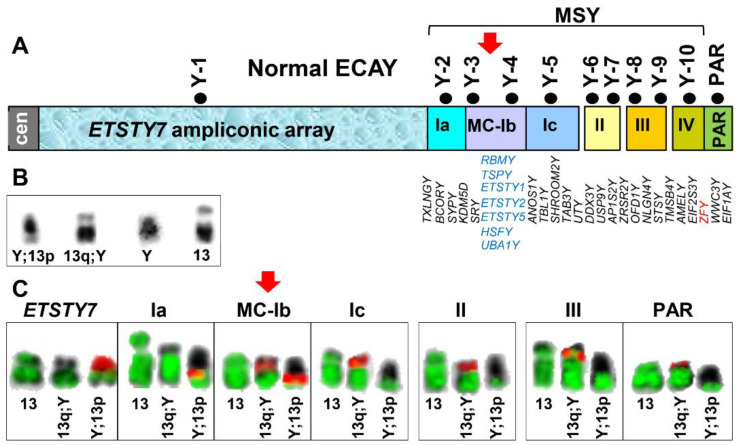
Determining the extent of genetic exchange between ECAY and ECA13. (**A**) ECAY sequence map [9] showing Y heterochromatin (*ETSTY7*), contigs I-IV and the PAR; contig I is divided into three regions: single-copy Ia, multicopy (MC) Ib, and single-copy Ic; contigs II–IV are single-copy; black dots with marker IDs above each region/contig correspond to the FISH markers used in this study (see Table 1); single copy gametologs are presented in map order below Y contigs in black font and sideways orientation; location of *ZFY* is highlighted in red font; contig MC-Ib multicopy genes are in blue font and horizontally stacked with no known map order; (**B**) inverted DAPI images of the derivative chromosomes Y;13p and 13q;Y, normal ECAY (Y) from a control horse, and normal ECA13 (13) (**C**) FISH results with ECAY markers (red) and ECA13 painting probe (green) representing different regions of the chromosome. Note that only ECAY multi-copy contig MC-Ib marker hybridizes to both aberrant chromosomes, thus marking the translocation breakpoint in ECAY (red arrows).

The first set of FISH experiments determined the overall extent of the genetic exchange between ECAY and ECA13. We selected from the horse Y chromosome sequence map [9] a set of markers representing the linear order of all the main regions in the horse Y chromosome, the proximal *ETSTY7* ampliconic array (Y heterochromatin), different MSY contigs which included the multicopy region in contig Ib and the PAR (Figure 1A, Table 1). In a series of FISH experiments, we co-hybridized individual Y markers or pooled two markers from the same contig with a microdissected ECA13 painting probe. The results showed that the cells of the Friesian stallion carry a normal ECA13 and two different derivative chromosomes designated as Y;13p and 13q;Y (Figure 1B). The derivative chromosome Y;13p was an acrocentric, which the proximal part corresponded to *ETSTY7* ampliconic array and contigs Ia and MC-Ib, whereas the distal portion corresponded to ECA13 (Figure 1B). The second derivative chromosome 13q;Y was a small sub-metacentric with the long arm corresponding to ECA13 and the short arm corresponding to the distal region of MSY and the PAR (Figure 1B). The only MSY region present on both derivative chromosomes was the multicopy contig Ib, suggesting that the translocation breakpoint in ECAY is in contig Ib. Since these experiments used the whole chromosome painting probe, it was not possible to determine the translocation breakpoint in ECA13.

**Figure 2:**
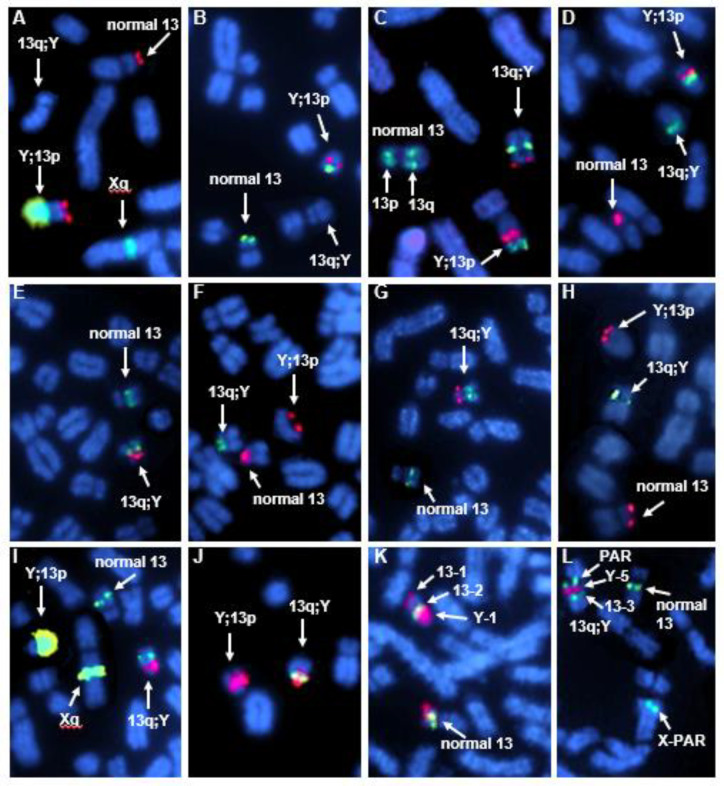
Partial metaphase spreads showing dual-color FISH results determining the size and orientation of translocated segments between ECAY and ECA13. (**A**) 13-1 red/Y-1 green; full metaphase spreads of H787 and a control male horse with the results of this FISH experiment are presented in Supplementary Figure S1; (**B**) 13-1 green/Y-2 red; (**C**) 13-2 green/13-3 green/Y-3 red; (**D**) 13-1 red/Y-4 green; (**E**) 13-3 green/Y-6 red; (**F**) 13-1 red/Y-7 green; (**G**) 13-3 green/Y-8 red; (**H**) 13-1 red/Y-9 green; (**I**) Y-1 green/Y-10 red/13-3 green; (**J**) Y-3 red/Y-5 green; (**K**) Y-1 red/13-2 green/13-1 red; (**L**) 13-3 green/Y-5red/PAR green.

Next, we co-hybridized pairwise or in 3-probe combinations ECA13p and ECA13q markers (Table 1) with the 11 ECAY markers (Table 1, Figure 1A). The FISH results confirmed but also refined the initial findings with the ECA13 painting probe. We showed that ECA13p was syntenic with the *ETSTY7* array and MSY contigs Ia and MC-Ib in derivative chromosome Y;13p (Figure 2A–D; Supplementary Figure S1), while ECA13q was syntenic with MSY contigs MC-Ib–IV and the PAR in derivative chromosome 13q;Y (Figure 2E,G,I,J). Based on this, we assigned the translocation breakpoint in ECA13 to the centromere. In line with the initial FISH results (Figure 1B), the translocation breakpoint in ECAY stayed in the multicopy region since both MC-Ib markers, Y-3, and Y-4 (Figure 1A, Table 1), provided hybridization signals on both derivative chromosomes (Figure 2C,D). However, because of the multicopy nature of these sequences, it was not possible to further narrow down the breakpoint in contig MC-Ib. This also means that we were not able to determine the location of the single copy equine *SRY* gene because it is embedded in the multicopy sequences in marker Y-3 (BAC 140M23, Table 1) [9].

**Figure 3:**
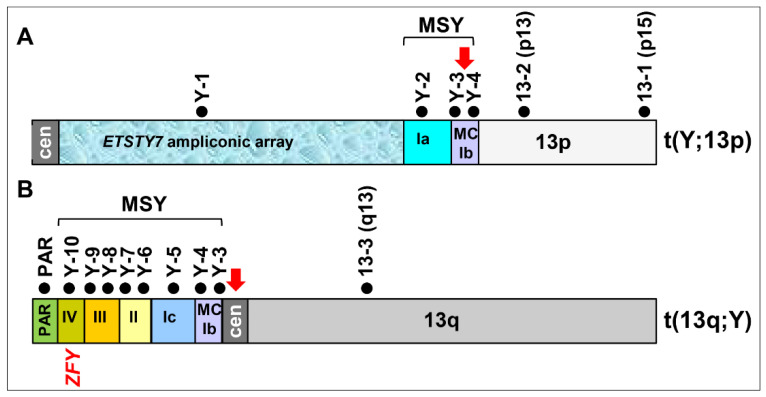
Schematic representation of the reciprocal translocation between ECAY and ECA13. (**A**) Aberrant chromosome Y;13p comprised of the proximal *ETSTY7* ampliconic array, MSY contigs Ia, part of MC-Ib and ECA13p; (**B**) aberrant chromosome 13q;Y comprised of ECA13q and the distal portion of MSY, including part of multicopy contig MC-Ib and single-copy contigs Ic, II, III, IV and PAR; black dots with marker IDs above each chromosome denote ECAY and ECA13 markers that were used for refined FISH analysis (see Table 1 and Figure 1 and Figure 2); red arrows indicate translocation breakpoints in ECAY contig MC-Ib and at ECA13 centromere; the location of *ZFY*, a candidate meiotic executioner gene is indicated.

Finally, we used dual-color FISH and combinations of three select markers to determine the orientation of reciprocally translocated segments of MSY and ECA13p (Figure 2K,L). We showed that in Y;13p, the ECA13p segment is attached to MSY contig MC-Ib by the proximal region with ECA13p15 remaining terminal in this derivative chromosome (Figure 2L and Figure 3A). Likewise, the terminal end of the short arm of the derivative chromosome 13q;Y corresponded to PAR with MSY contigs IV-III-II-Ic-MC-Ib, located proximally (Figure 2K and Figure 3B).

A summary of the ECAY-ECA13 reciprocal translocation is presented in Figure 3. The results show that the heterochromatic (*ETSTY7* array) portion of ECAY together with a small single copy and partial multicopy region have become syntenic with the short arm of ECA13 (Figure 3A), whereas most of the single copy MSY together with the partial multicopy region and the PAR have become syntenic with the long arm of ECA13 (Figure 3B). Translocation breakpoints were assigned to the MSY multicopy region and ECA13 centromere.

### 3.2. Copy Number Analysis of Horse MSY Multicopy Genes

After revealing that MSY multicopy contig MC-Ib (Figure 1A) sequences were present in both derivative chromosomes and that the translocation breakpoint in the Y chromosome was in the multicopy region, we further studied this region for gene copy number (CN) variation to see whether the translocation affected the CN of known MSY multicopy genes. We determined the absolute copy numbers of seven MSY contig Ib multicopy and testis-specific genes (*TSPY*, *RBMY*, *ETSTY1*, *ETSTY2*, *ETSTY5*, *HSFY*, and *UBA1Y*) [9] and *SRY* in the Friesian stallion and the chromosomally normal control males. The latter also included the DNA donor for horse MSY reference assembly, a Thoroughbred stallion Bravo. The results showed that CNs of four multicopy genes (*RBMY*, *ETSTY2*, *HSFY,* and *UBA1Y*) and the *SRY* were not statistically different between the Friesian stallion and the controls (Table 3). However, CNs of a protein coding gene *TSPY* and two testis-specific transcripts, *ETSTY1* and *ETSTY5*, were significantly (*p* < 0.05) lower in the Friesian stallion, with the most significant (*p* = 0.004) CN reduction for *ETSTY5* (Table 3).

## 4. Discussion

Here we presented a detailed molecular cytogenetic analysis of a reciprocal translocation between ECAY and ECA13 in an infertile Friesian stallion. This is the first and so far only case of Y-autosome translocation and one of the very few cytogenetically detectable Y chromosome structural rearrangements reported in horses (see [25]). Among the latter are two intersex horses with mosaicism for isochromosome Y [26,27], a few cases of XY females with large Y chromosome deletions [28] and a pony with abnormal external genitalia (no penis) and large deletion of the *ETSTY7* ampliconic array, also known as Y heterochromatin [25]. The low number of reported cases may be because structural rearrangements of the Y chromosome are rare in horses, though it is more likely that they are not discovered because breeding horses are not subject to routine cytogenetic screening. It is also possible that due to the small size of the Y chromosome, structural rearrangements may easily remain undetected during conventional cytogenetic analysis. For example, in the present case of ECAY-ECA13 reciprocal translocation, the derivative chromosome Y;13p could easily pass for a normal Y chromosome, the derivative chromosome 13q;Y was very similar in size, morphology, and DAPI-banding to the normal ECA13 (Figure 1B) [8].

The clinical characterization of this case was published recently [8]. However, despite the detailed reproductive workup and a logical conclusion that the observed azoospermia was the likely consequence of the Y-autosome translocation, the study lacked a clear cytogenetic and molecular explanation. Meiotic studies of analogous Y-autosomal translocations in men with nonobstructive azoospermia have proposed several explanations for impaired spermatogenesis. These include disruption of autosome and sex chromosome pairing in meiosis, thereby leading to impaired sperm production, and degeneration of spermatocytes and apoptosis [29]. Similar disturbances in meiotic chromosome behavior have been documented in pigs showing that Y-autosome translocations result in increased instances of abnormal pachytene synapsis leading to the cell cycle arrest at the stage of primary spermatocytes [2,3]. In the present case, it was not possible to obtain testicular material for meiotic studies. Testis histopathology, conducted by Ruiz et al. [8], showed only that seminiferous tubules of the Friesian stallion had reduced diameter and contained Sertoli cells only, indicating early spermatogenic arrest. However, we have no information whether spermatogenesis proceeded through prophase I, or even to meiosis I. Therefore, any discussion about the meiotic behavior and possible synaptic configurations between the normal and aberrant chromosomes in this case, remain too speculative. Furthermore, as shown by meiotic studies of Y-autosome translocation in pigs [5], chromosome configurations and the extent of synapses tend to vary between cells.

However, regarding the phenotypic effect of Y-autosome translocations, it is perhaps even more important to understand how different meiotic configurations can impact MSCI and the regulation of genes residing in the chromosomes involved. Here again, due to the lack of our own data, we must rely on immunogenetic and gene expression studies of meiosis of Y-autosome translocations in other species. Both human [13,30] and pig [2,3,5] studies show that Y-autosome translocations disturb MSCI and the formation of sex body in meiosis prophase I. Depending on the synaptic configurations formed in a particular cell, the sex body, which is immunogenetically visualized by the accumulation of histone variant γH2AX [31], can spread from sex chromosomes to the autosome and silence autosomal genes [2,5]. Alternatively, there may be cells with no sex body formation and thus, no MSCI [3,5,13,32]. For example, in a study of Y-SSC13 translocation in an azoospermic boar, a sex body was found in approximately 50% of cells [5]. In either scenario, i.e., sex body spreading over autosomes vs. no sex body at all, Y-autosome translocations pose a meiotic conflict between the necessary transcriptional activity of autosomal genes and the obligatory silencing of the sex chromosomes. Therefore, one hypothesis trying to explain the meiotic arrest and azoospermia in these cases is that transcriptional silencing of certain regions in the autosomal genome leads to meiotic arrest [13,30,33]. However, the autosomes involved in translocations with the Y chromosome in humans, pigs, cattle and in the present equine case are not homologous, thus not carrying orthologous genes. For example, reported Y-autosome translocations in humans involve all autosomes except HSA20 [34,35], pig cases involve autosomes SSC1 [3], SSC13 [3,5], and SSC14 [2], and cattle cases just two autosomes, BTA9 [7] and BTA21 [6]. Therefore, if autosomal factors are responsible for meiotic arrest in these cases, it must be due to transcriptional silencing of autosomal genes per se and not due to specific genes or regions.

On the other hand, while Y-autosome translocations involve different and non-homologous autosomes in different species, a common denominator for all cases is the involvement of the Y chromosome. Therefore, and as shown by several studies [3,4,5,17,36], a more plausible explanation for meiotic arrest is the failure to properly inactivate sex chromosomes, particularly the Y chromosome. This is in line with a recently presented theory about “the persistent Y” and “meiotic executioner genes” [37]. The theory provides a novel mechanistic explanation of why, despite of millions of years of degenerate evolution, the eutherian Y chromosome persists. The theory postulates that the Y-linked meiotic executioner genes are necessary for successful meiosis, but must also be subjected to MSCI, and regulate their own silencing. Ectopic expression of these genes during the silencing window in cases of Y-autosome translocations will result in fatal meiotic arrest [17,36]. In turn, meiotic arrest prevents the transmission of translocations and as a result, the Y chromosome persists [37]. The “persistent Y theory” proposes *ZFY* as the most likely “meiotic executioner” gene because, firstly, it is among the few genes found in all eutherian Y chromosomes [9,10,38], and secondly, it is the only conserved eutherian Y gene whose inappropriate expression through the MSCI window is pachytene lethal [39]. In contrast, the ectopic expression of other conserved eutherian Y genes such as *RBMY, UTY, DDX3Y,* and *SRY* does not induce pachytene arrest [36,37]. In the present equine case, there was no possibility to study meiosis cytogenetically or for gene expression. Nevertheless, as we determined the translocation breakpoints and the genetic content of the two derivative chromosomes (Figure 3), we can show that the derivative chromosome with the largest autosomal portion, 13q;Y, also carried the *ZFY* gene (Figure 3B). This allows us to theorize that, if *ZFY* is the true meiotic executioner gene and if the derivative 13q;Y synapsed in meiosis with normal 13q, *ZFY* may have escaped MSCI leading to meiotic arrest and azoospermia.

The theory about meiotic executioner genes with *ZFY* as the primary candidate [37], also explains why some cases of Y-autosome translocations in animals [6] and humans [11,12] do not result in meiotic arrest. For example, the two published Y-autosome translocations in cattle [6,7] have contrasting phenotypes. The case with azoospermia [7] had a reciprocal translocation between Y and BTA9, so that the two derivative chromosomes comprised parts of the Y and large portions of BTA9. While the authors proposed that azoospermia was caused by the production of unbalanced gametes due to the formation of quadrivalent configurations in meiosis [7], an alternative explanation is the failure to silence the *ZFY* gene in BTAYp [40] which was translocated to the distal half of BTA9. In contrast, the Y-autosome translocation in a reproductively normal bull [6] was non-reciprocal, so that one derivative chromosome comprised of BTA21 and BTAYq, while BTAYp with *ZFY* and PAR [40] remained a separate chromosome and could easily undergo MSCI. Likewise, in humans where *ZFY* is located in HSAYp [41], non-reciprocal translocations of HSAYq to an autosome do not affect the phenotype or fertility [11,12], while balanced reciprocal Y-autosome translocations involve euchromatic portions of the Y, including HSAYp, result in oligozoospermia or azoospermia [11,13,14]. Therefore, to evaluate the genetic and phenotypic consequences of Y-autosome translocations, one must not only examine the cytogenetic features of the aberration but also must have knowledge about the organization of the Y chromosome of the species in question.

Lastly, we asked whether the presented case of a reciprocal translocation between ECAY and ECA13 was balanced. For this, we first tried to pinpoint the translocation breakpoint. This appeared to be difficult because the breakpoint was in the multicopy region of Y and BAC-FISH produced hybridization signals in both derivative chromosomes (Figure 1C and Figure 2C,D), thus confounding the precise demarcation of the breakpoint. To obtain more information about the breakpoint region, we evaluated copy numbers (CN) of seven known ECAY multicopy genes [9] and the *SRY* (all located in contig MC-Ib; Figure 1A), and showed significant CN reduction for three genes/transcripts: *TSPY*, *ETSTY1,* and *ETSTY5* (Table 3). These findings may suggest that the translocation was accompanied by the loss of some multicopy sequences and was, thus, not balanced. Though it is also possible that the observed CN variation was specific to the individual or the breed and needs further investigation. The functional significance of the *TSPY* copy number variation in stallions or other species is not known, though the gene has been associated with male fertility in cattle [42] and humans [43]. Possible functions or the protein coding potential of equine testis-specific transcripts, *ETSTY1* and *ETSTY5*, are yet not known [9].

## 5. Conclusions

In summary, molecular cytogenetic characterization, and copy number analysis of the first Y-autosome reciprocal translocation in horses adds a new case to equine clinical cytogenetics but also poses questions about the behavior of derivative chromosomes and the regulation of Y chromosome genes in male meiosis.

## Figures and Tables

**Table 1:** Information about all BACs used for FISH in this study.

Marker ID	CH241 BAC	Cytogenetic Location	Location in EquCab3 or ECAY BAC Contig Map	Reference Marker	Reference
Y-1	069E11	Yq proximal 2/3	Y and Xq heterochromatin	*ETSTY7* ampliconic array	[9]
Y-2	022P7	Yqdistal	Y Contig Ia, single copy	*KDM5D*	[9]
Y-3	140M23	Yqdistal	Y Contig Ib, multicopy (MC)	*SRY*	[9]
Y-4	017D15	Yqdistal	Y Contig Ib, multicopy (MC)	*TSPY*	[9]
Y-5	090G18	Yqdistal	Y Contig Ic, single copy	n/a	[9]
Y-6	112E12	Yqdistal	Y Contig II, single copy	*NLGN4Y*	[9]
Y-7	011B8	Yqdistal	Y Contig II, single copy	n/a	[9]
Y-8	125H6	Yqdistal	Y Contig III, single copy	*TMSB4Y*	[9]
Y-9	102J15	Yqdistal	Y Contig III, single copy	*TMSB4Y*	[9]
Y-10	106F1	Yqdistal	Y Contig IV single copy	*ZFY*	[9]
PAR	194E12	Xpter/Yqter	chrX:3945-246,703	PLCXD1	[20]
13-1	078E13	13p15	chr13:5,913,678-6,105,097	*GPER1*	This study
13-2	060D24	13p13	chr13:11,481,169-11,662,804	*ELN*	This study
13-3	158P20	13q12	chr13:18,059,033-18,254,381	LEX041	[19]

**Table 2:** Digital droplet assays for copy number analysis of MSY multicopy genes and *SRY*.

Gene	Forward Primer 5′–3′	Reverse Primer 5′–3′	Probe Sequence 5′–3′
*TSPY*	CATAGTGGAGGAAGAGGATGAAA	GGCAATGGTTTAACCCTGAAA	CTCTTTCTGGGAGACCTGCCCTTT
*SRY*	TTCTGTGATCTATGCTGGCG	TTACCCTCCGGACTTTCTCA	AACAGGGACTCTGCCGCCACCA
*RBMY*	GAAGCTCCACAACTTGAGGT	CTCTGACCTATGATGGAAGCA	TGTCTGCCACCATGCTCACGACCA
*ETSTY1*	GACGGACGACCTTGTGTT	ACGCTCACAGATGACAGTAG	TGTCCCGGCCACCTCAGGGC
*ETSTY2*	TTGTTGTTAGGCTACCTGGC	AAGGGCAAACCATAACCTCC	TGGGCAAGCTTCTCCATGGTTGCTGCA
*ETSTY5*	GAGGCAGGTACTTCGTTACC	TCACTCACAAAGTCAACGCT	TGCCGTGAGCTTGAGGGCGAA
*UBA1Y*	TTTCTGTTGTCTGGACGGAG	CTCCACGGATGTAGTCAGAG	AGCAGAGGCCTCCTGTGTCTGAGCT
*HSFY*	AGGCTTTCTCCACTGGTTTC	GAGGCTGTCCCGAACTTTTA	CCCCTGCTCTAAAGTGCTTCCTGTCG
*MYOZ1*	GACTTTCCAGATGCCCAAGT	ACCAGAACCTCTCCAACAGGCCTTCT	GCTCCTCTGTTTCTCCATCC

**Table 3:** Copy number (CN) analysis of seven MSY multicopy genes and the single copy *SRY* gene in the Friesian stallion (H787) and 16 control males.

Sample ID	*TSPY*	*ETSTY1*	*ETSTY2*	*ETSTY5*	*SRY*	*RBMY*	*HSFY*	*UBA1Y*
H787	5.6	1.9	3.8	2.6	0.8	1.9	0.9	3.1
23346	9.1	3.6	5.7	5.3	1	1.5	0.9	3.8
23348	8.2	7.4	5.7	4.8	1	2.3	0.9	3.5
70858	9.7	4.1	4.2	4.1	0.8	2	0.9	3.2
70980	10.8	3.9	5.1	4.4	0.9	1.7	0.9	3.8
70981	11.3	3.8	4.6	4.4	0.8	1.7	1.1	3
73901	8.6	3.9	4.1	3.9	1	2	1	3
74413	8.2	3.9	4.3	4.3	0.9	1.9	1	3.6
74836	8.2	3.7	4.6	4	0.8	1.7	1.2	3.3
74837	8.6	3.9	4.5	3.7	0.7	1.9	1	3.8
75052	9.2	3.6	4.6	4.2	0.9	2	1	3.7
TR007	9	4.2	3.8	4.1	1	1.9	1	3.9
TR008	9.5	4	3.7	4.1	0.9	2.1	1	4.3
TR009	8.2	3.9	3.8	3.8	0.9	2	1	4
H061	8	4.1	4.5	3.6	0.8	1.9	1.1	1.8
H294	13.3	3.8	5.4	4.4	0.8	1.7	1.1	3.3
Bravo	8.5	4.9	5.6	3.6	0.9	2	1.1	1.5
*p*-value	0.0249 *	0.0293 *	0.2494	0.0038 **	0.4003	0.9756	0.2365	0.7583

CNs are rounded to the nearest tenth and CN values in red font indicate the lowest CN for each gene; statistically significant *p*-values are denoted as (*) *p* < 0.05 or (**) *p* < 0.01; sample ID Bravo is the DNA donor for horse MSY reference assembly.

## Data Availability

Not applicable.

## Supplementary Materials

The following are available online at https://www.mdpi.com/article/10.3390/genes12121892/s1, Figure S1: Three versions of the same metaphase spread of the Friesian stallion (A–C) and a normal control male horse (D–F) showing FISH results with probes Y-1 (green) and 13-1 (red). Images A and D show FISH signals as green and red, and chromosomes as blue (DAPI); images B and E show FISH signals as green and red, and chromosomes as inverted DAPI, and images C and F show only chromosomes as inverted DAPI. Images A–C correspond to the partial metaphase in Figure 2A.
