# Supplementary material for: Molecular Cytogenetic and Y Copy Number Analysis of a Reciprocal ECAY-ECA13 Translocation in a Stallion with Complete Meiotic Arrest
Source: Genes (Basel). 2021 Nov 26;12(12):1892. doi: 10.3390/genes12121892 (PMC8701272; doi:10.3390/genes12121892)
Supplement: Supplementary file 1 [file genes-12-01892-s001.zip › genes-1463874-supplementary.pdf]

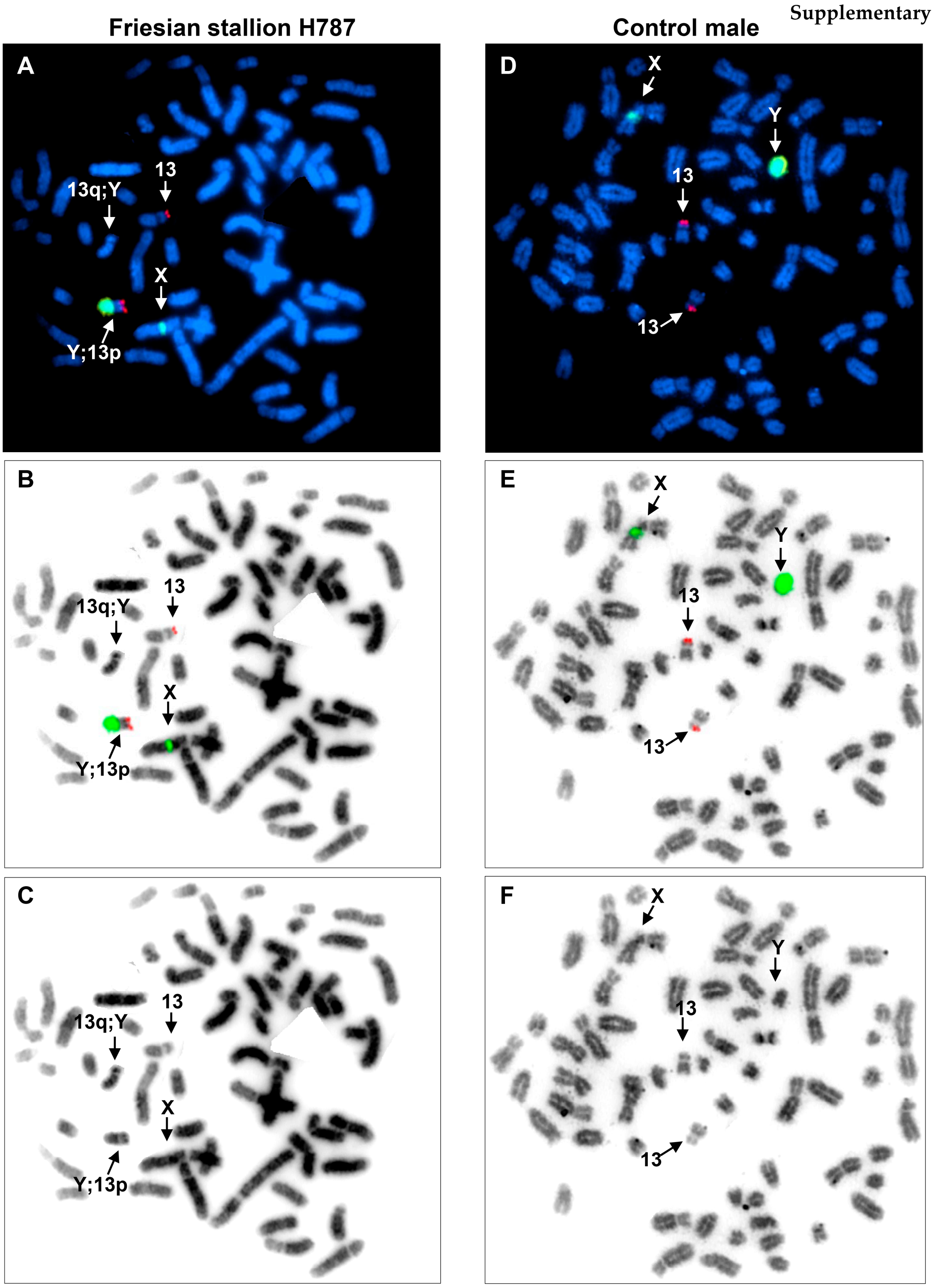

Supplementary Figure S1. Three versions of the same **m**etaphase spread of the Friesian stallion (A-C) and a normal control male horse (D-F) showing FISH results with probes Y-1 (green) and 13-1 (red). Images A and D show FISH signals as green and red, and chromosomes as blue (DAPI); images B and E show FISH signals as green and red, and chromosomes as inverted DAPI, and images C and F show only chromosomes as inverted DAPI. Images A-C correspond to the partial metaphase in Figure 2A
